# Supplementary material for: RNA Sequencing Analysis and Verification of Paeonia ostii ‘Fengdan’ CuZn Superoxide Dismutase (PoSOD) Genes in Root Development
Source: Plants (Basel). 2024 Jan 31;13(3):421. doi: 10.3390/plants13030421 (PMC10856844; doi:10.3390/plants13030421)
Supplement: Supplementary file 1 [file plants-13-00421-s001.zip › Supplementary Figure.pdf]

Supplementary Data

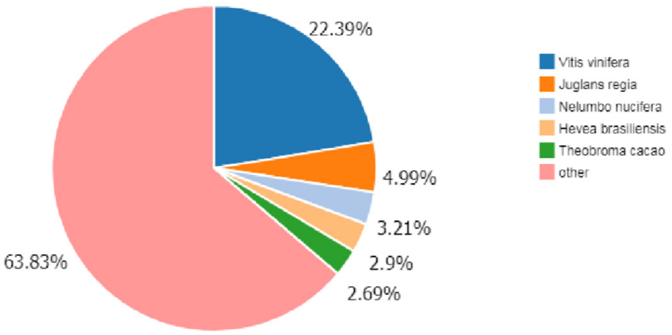

Figure S1. Similarity distribution blast to NR annotation of the tree peony.

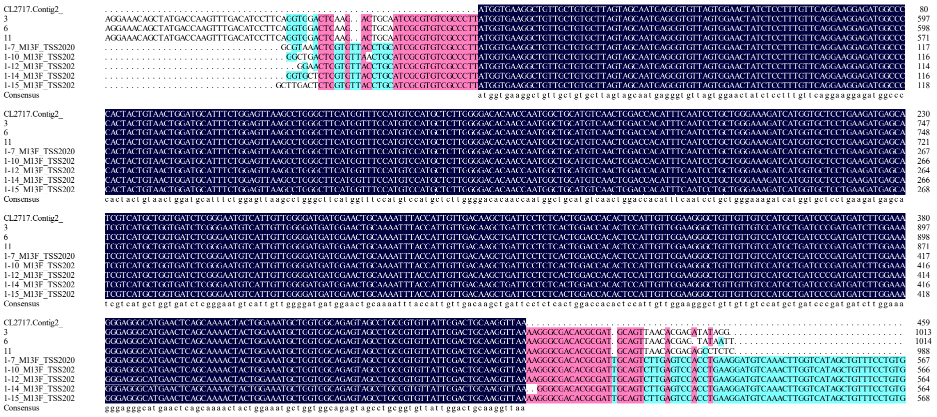

Figure S2. The sequence alignment results of the *PoSOD* transcriptome sequence and sequencing.

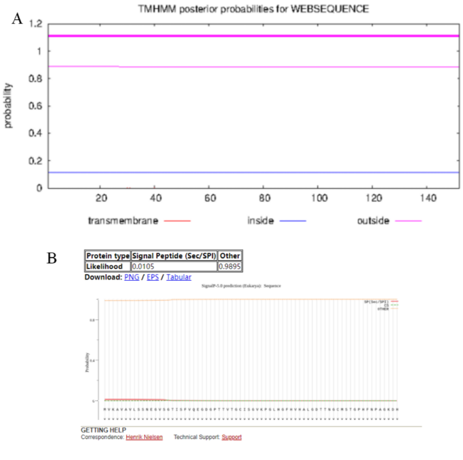

Figure S3. Transmembrane domain and signal peptide prediction of PoSOD.

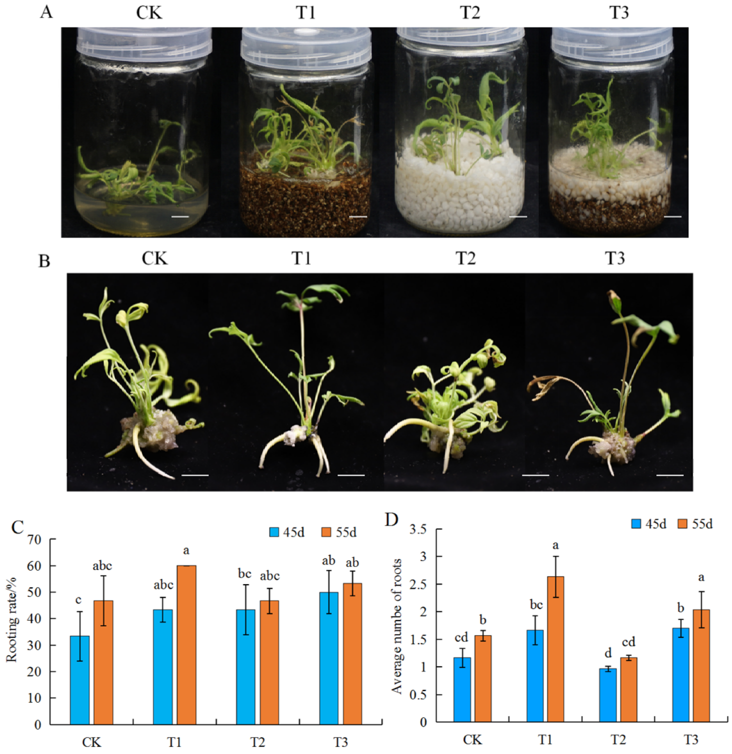

Figure S4. Effects of different substrate treatments including CK, vermiculite (T1), pyridine (T2), vermiculite : pyridine = 1:1 (T3) in tree peony tube plantle. (A)(B) Phenotype of tube plantlets treated with CK, T1, T2, T3 after 55d. (C) The rooting rate, (D) Average number of roots. Different letters represent significant differences between samples by Duncan's test, $P < 0.05$. Scale bar: 1cm.

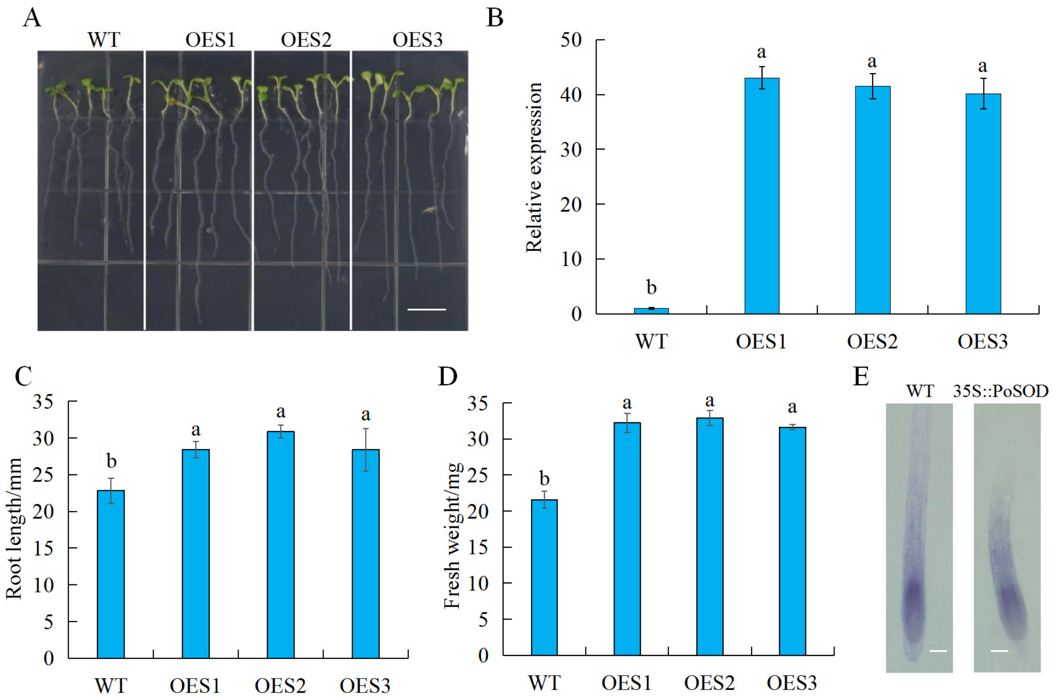

Figure S5. Phenotypic analysis of PRs in transgenic *PoSOD Arabidopsis*. (A) Phenotype of seedlings grown in 1/2 MS medium for 7 d after germination, scale bar: 1cm. (B) Relative expression of *PoSOD*. (C) Root length. (D) Fresh weight. (E) Nitroblue tetrazolium (NBT) staining, scale bar: 100 μM. Different letters represent significant differences between samples by Duncan's test, $P < 0.05$.

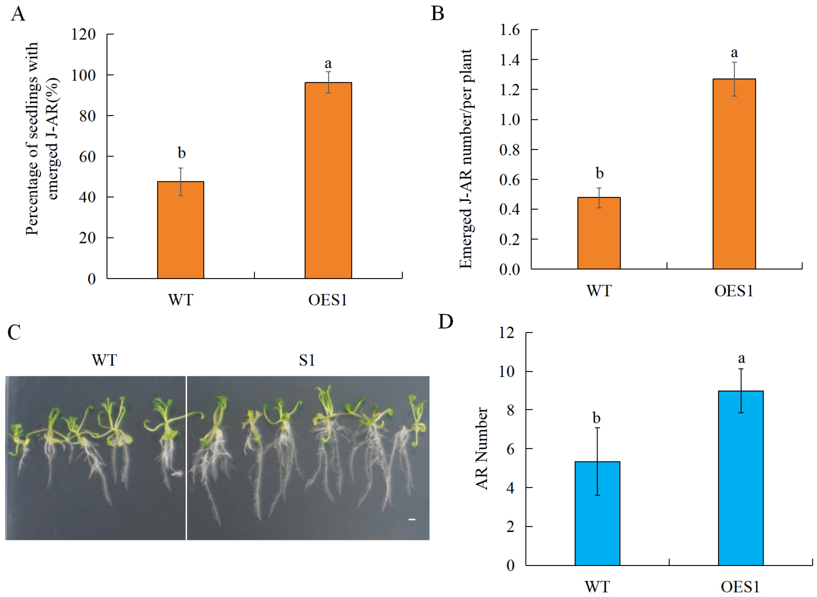

Figure S6. Phenotypic analysis of ARs in transgenic *PoSOD* in *Arabidopsis*. (A) Percentage of seedlings with emerged J-AR (B) Emerged J-AR number of per plant under low light conditions. (C) Representative figure of AR after treating the rootless of WT and S1 with 10 mg • L^{-1} IBA, scale bar: 1cm. (D) The statistics of AR number. Different letters represent significant differences between samples by Duncan’ s test, $P < 0.05$.

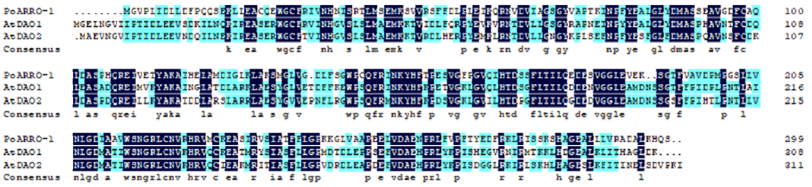

Figure S7. Multiple alignment of amino acid sequences for PoARRO-1 (GenBank: KJ620008), AtDAO1 (AT1G14130) and AtDAO2 (AT1G14120).

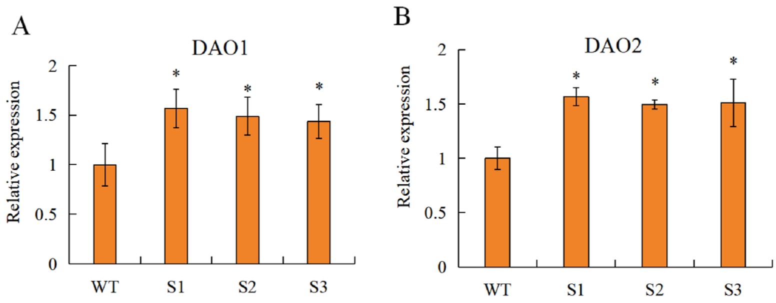

Figure S8. The expressions of DAO1 (A) and DAO2 (B) of WT and transgenic *Arabidopsis* after overexpression of *PoSOD*. Different letters represent significant differences between samples by Duncan’ s test, $P < 0.05$.

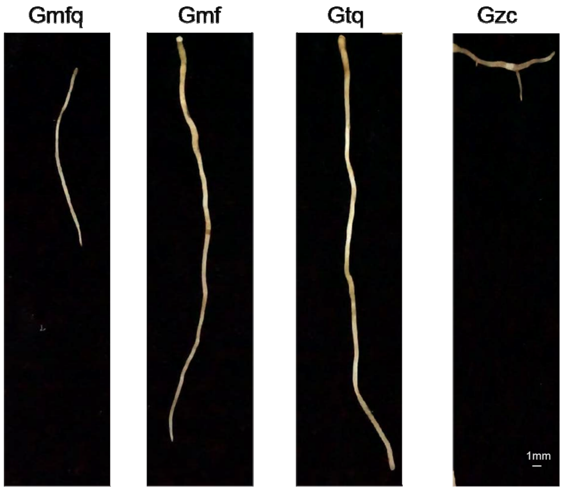

Figure S9. Four periods of adventitious root development in the tree peony.
