# Supplementary figures and images for: RNA Sequencing Analysis and Verification of Paeonia ostii ‘Fengdan’ CuZn Superoxide Dismutase (PoSOD) Genes in Root Development
Source: Plants (Basel). 2024 Jan 31;13(3):421. doi: 10.3390/plants13030421 (PMC10856844; doi:10.3390/plants13030421)

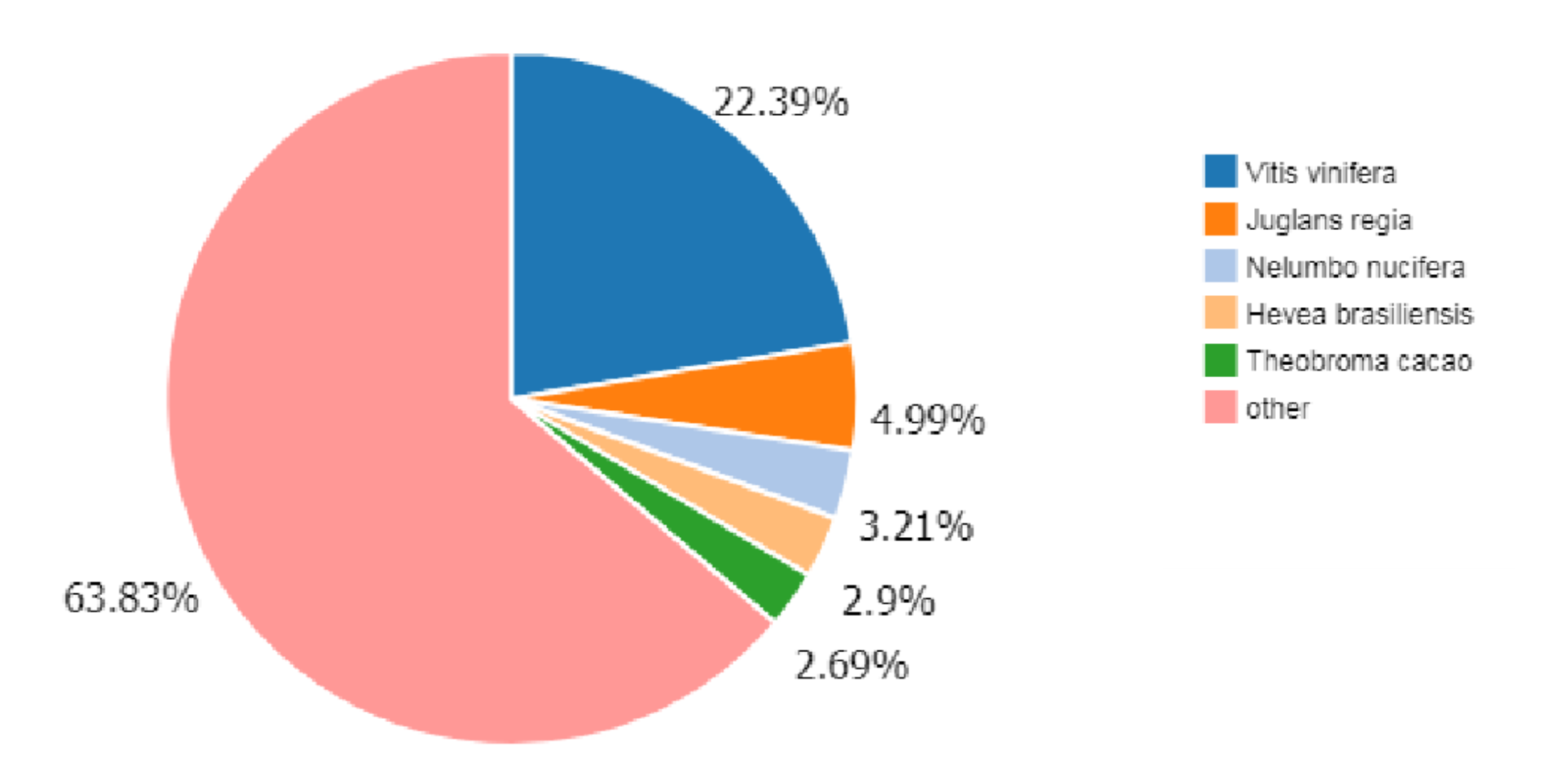

Supplement: Supplementary file 1 [file plants-13-00421-s001.zip › Figure S1.png]

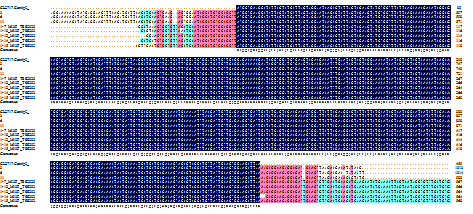

Supplement: Supplementary file 1 [file plants-13-00421-s001.zip › Figure S2.png]

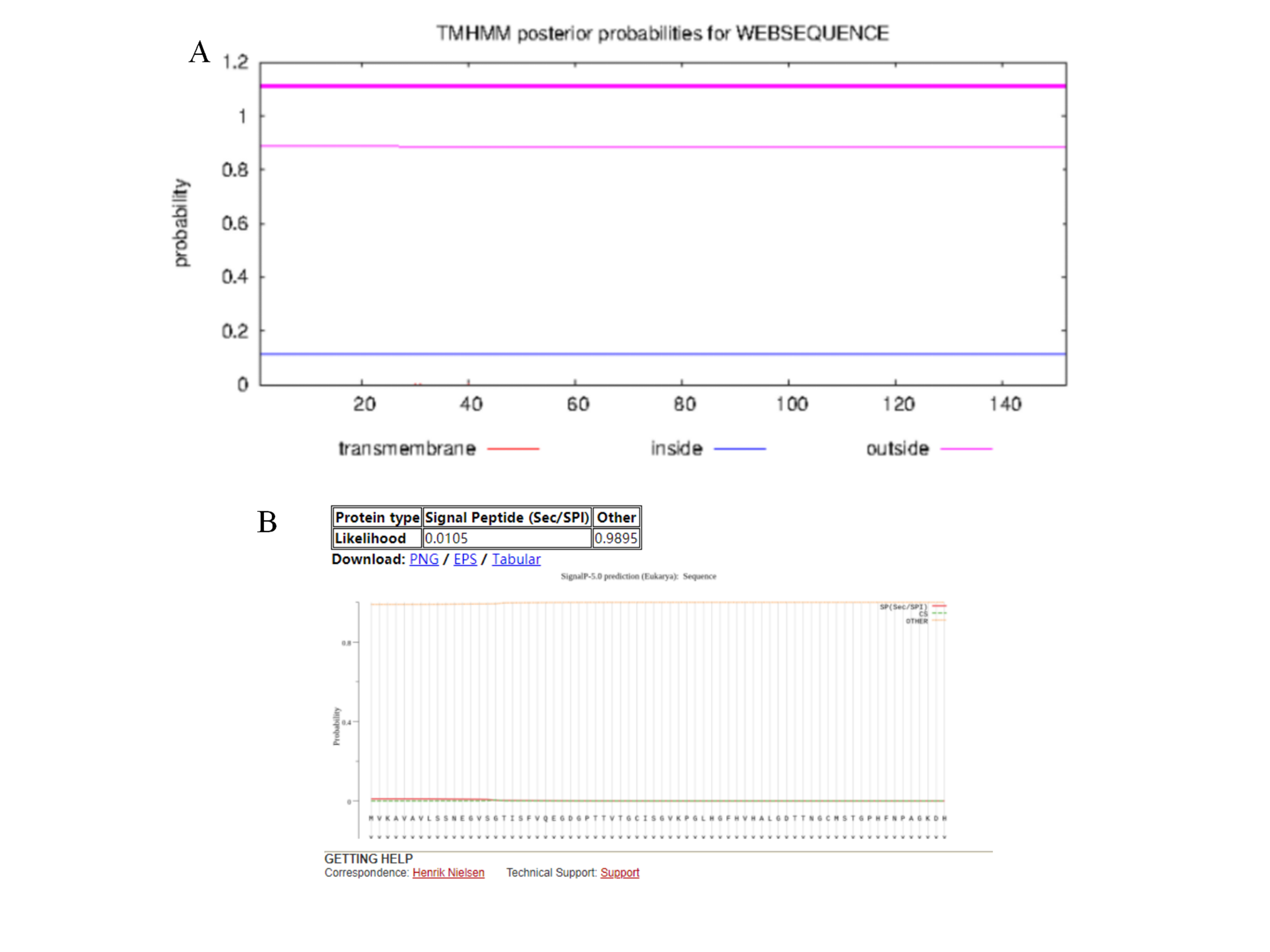

Supplement: Supplementary file 1 [file plants-13-00421-s001.zip › Figure S3.png]

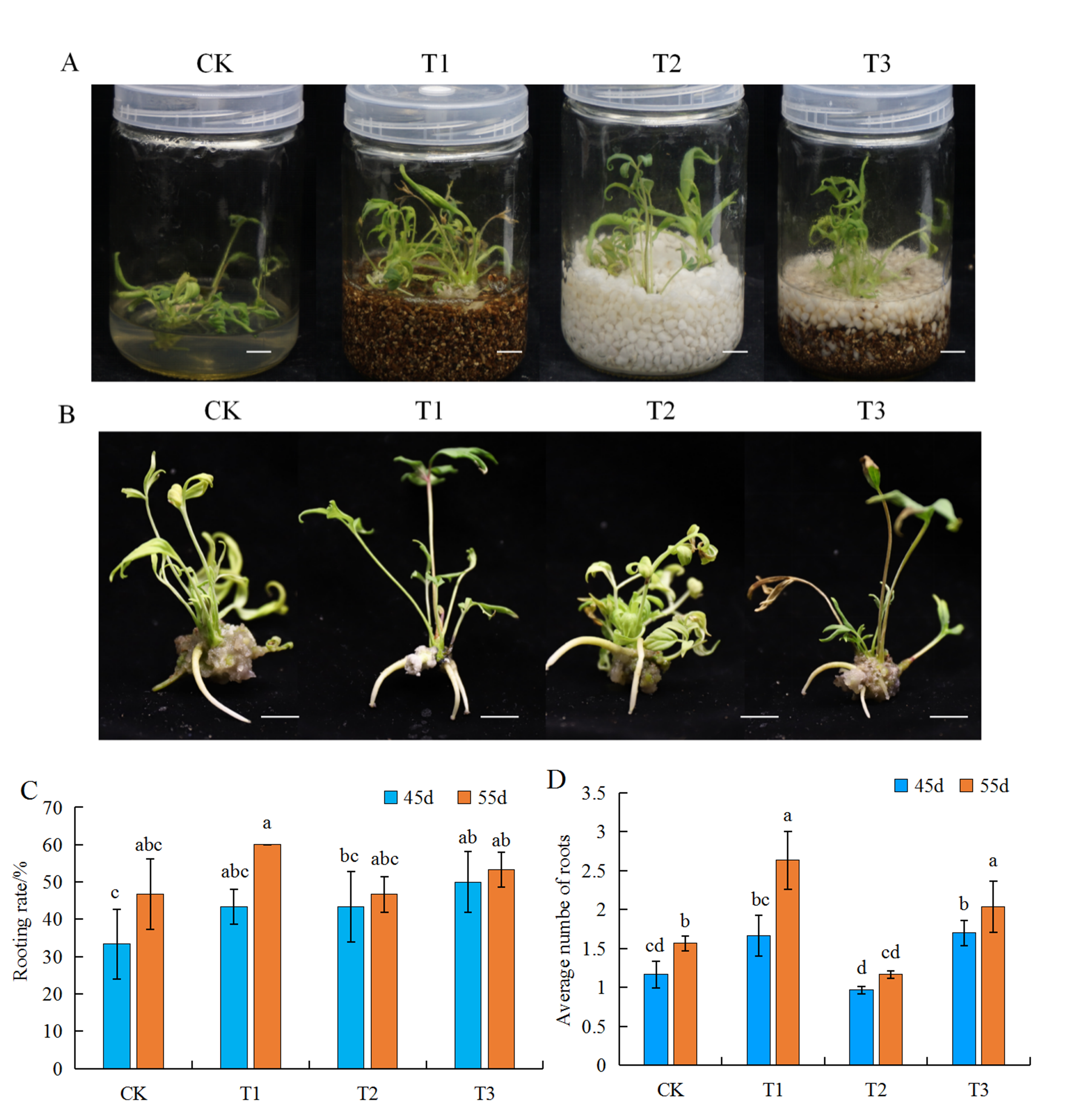

Supplement: Supplementary file 1 [file plants-13-00421-s001.zip › Figure S4.png]

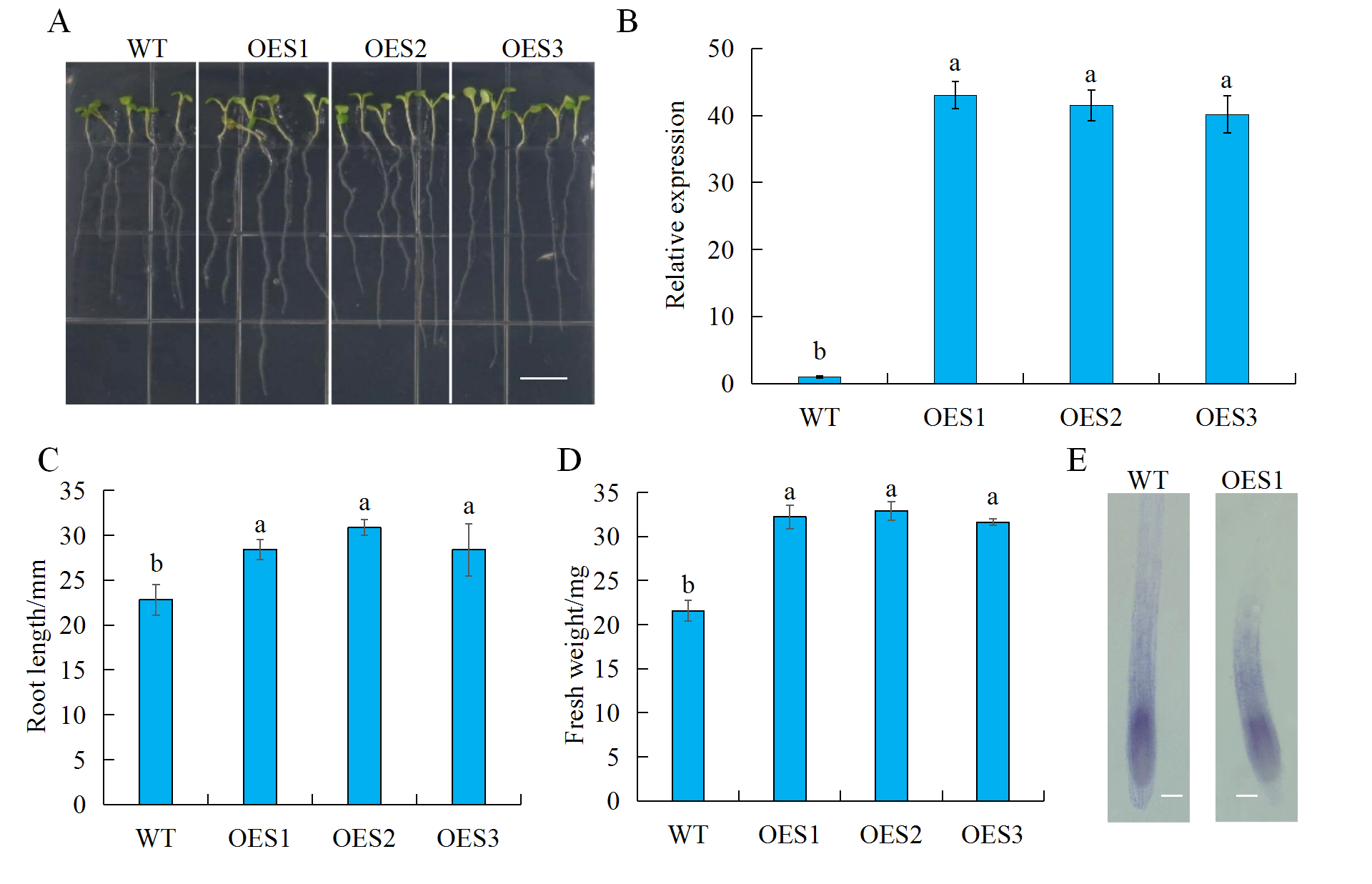

Supplement: Supplementary file 1 [file plants-13-00421-s001.zip › Figure S5.png]

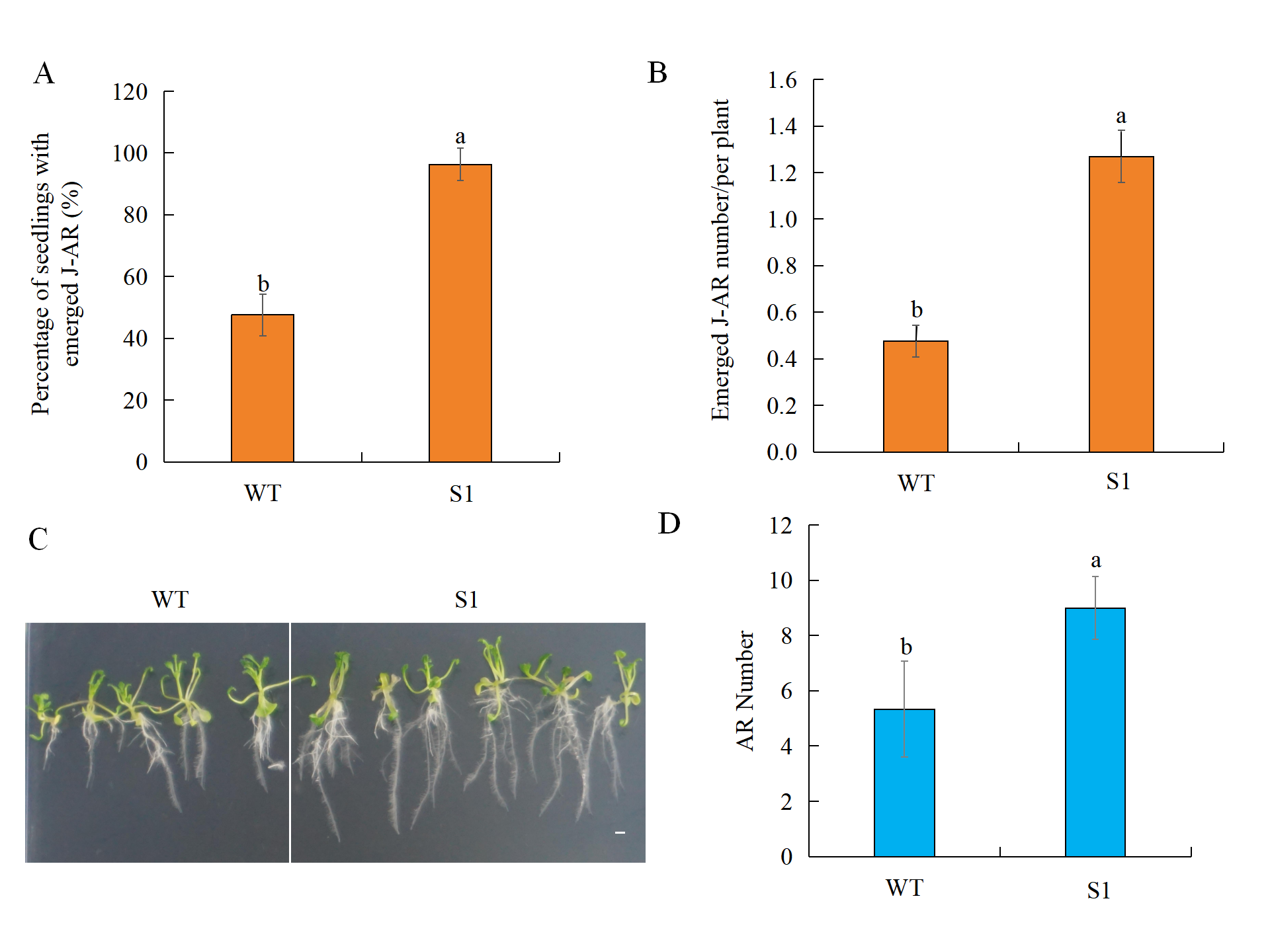

Supplement: Supplementary file 1 [file plants-13-00421-s001.zip › Figure S6.png]

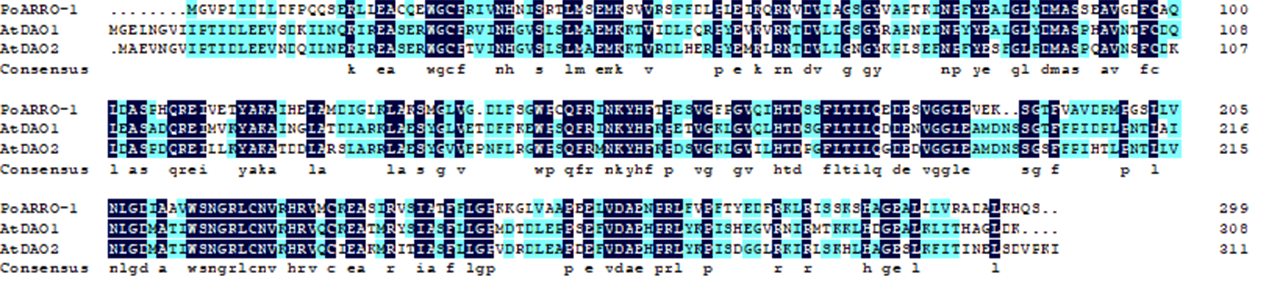

Supplement: Supplementary file 1 [file plants-13-00421-s001.zip › Figure S7.png]

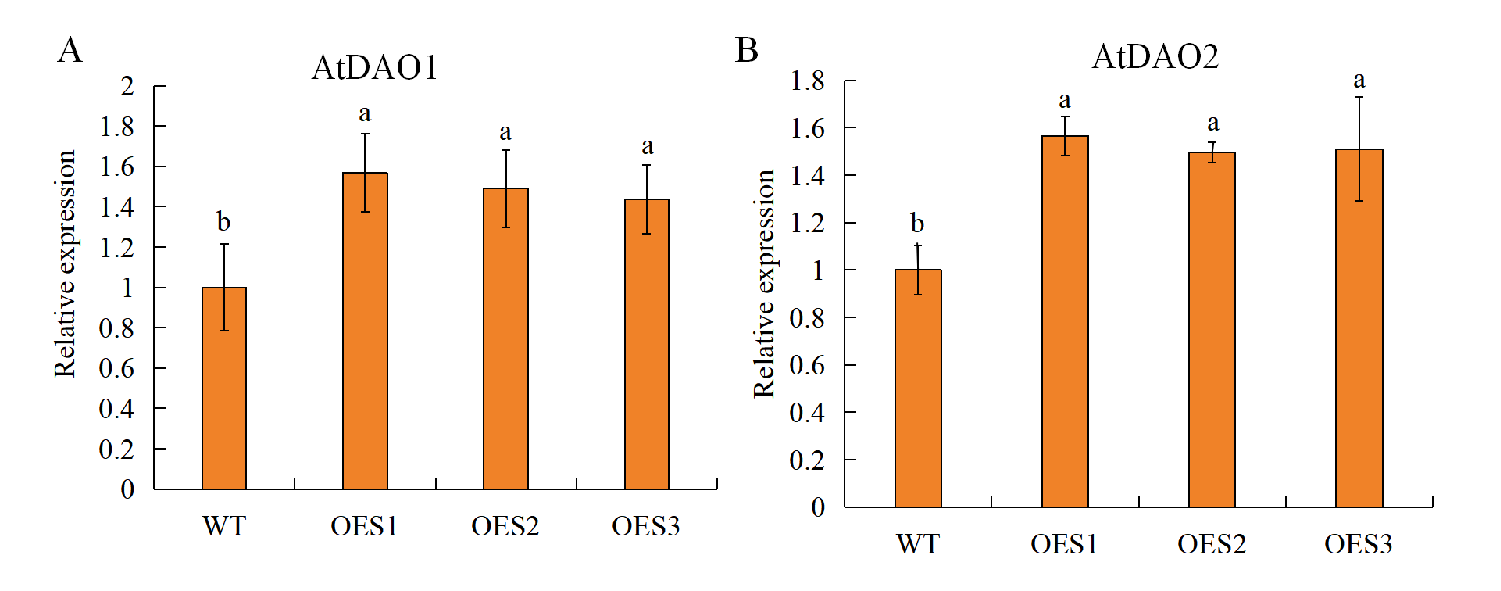

Supplement: Supplementary file 1 [file plants-13-00421-s001.zip › Figure S8.png]

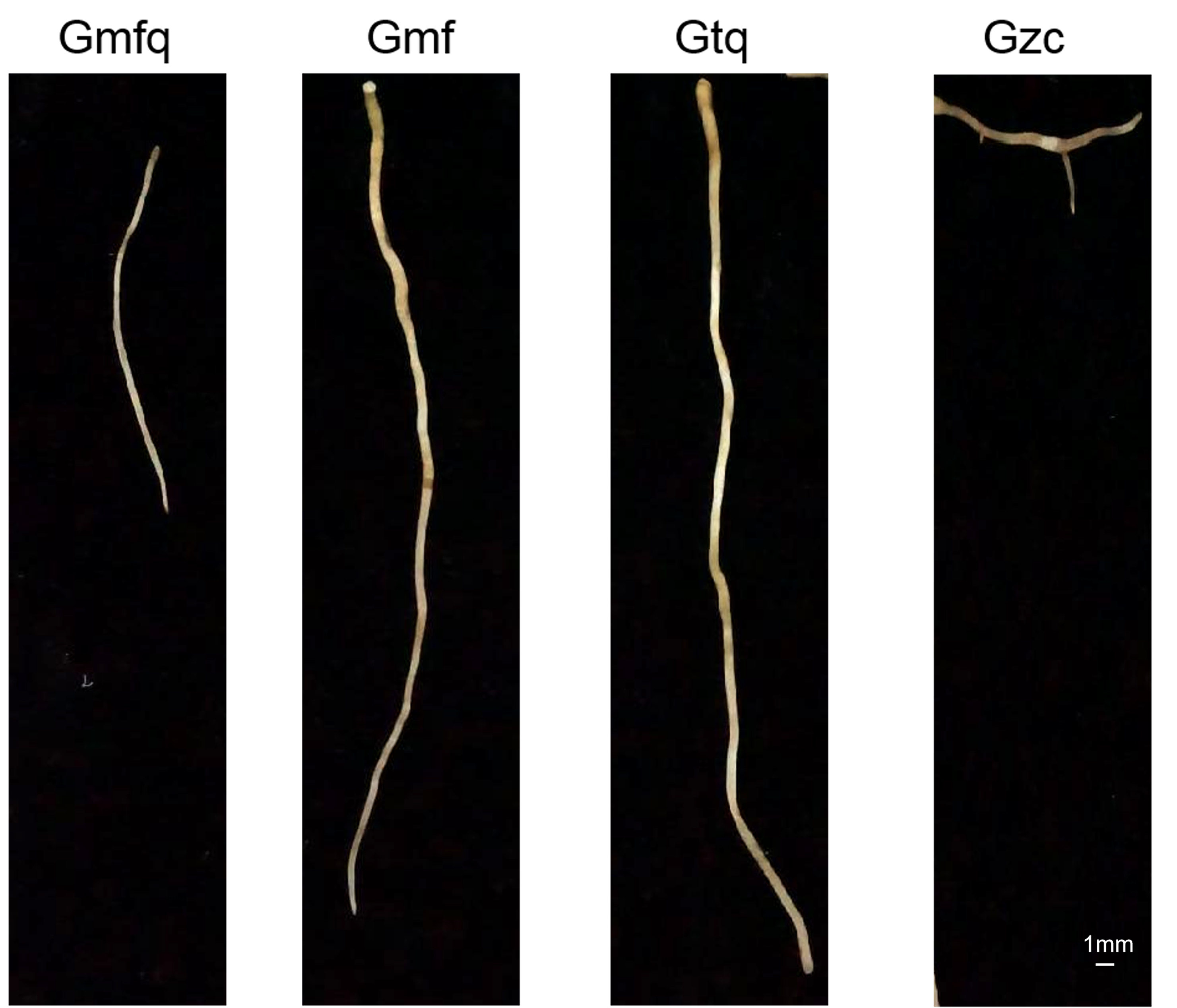

Supplement: Supplementary file 1 [file plants-13-00421-s001.zip › Figure S9.png]
